# Related quality of life questionnaire specific to dysthyroid ophthalmopathy evaluated in a population of patients with Graves’ disease

**DOI:** 10.1590/2359-3997000000252

**Published:** 2017-02-01

**Authors:** Laura Carolina Delfino, Anabela Zunino, Verónica Sapia, María del Carmen Silva Croome, Verónica Ilera, Alicia Teresa Gauna

**Affiliations:** 1 Hospital de Agudos J. M. Ramos Mejía Ciudad Autónoma de Buenos Aires Argentina División Endocrinología, Hospital de Agudos J. M. Ramos Mejía, Ciudad Autónoma de Buenos Aires, Argentina; 2 Hospital de Agudos J. M. Ramos Mejía Ciudad Autónoma de Buenos Aires Argentina Servicio de Oftalmología, Hospital de Agudos J. M. Ramos Mejía, Ciudad Autónoma de Buenos Aires, Argentina

**Keywords:** Graves ophthalmopathy, Graves’ disease, quality of life, orbitopathy, thyroid autoimmunity

## Abstract

**Objective:**

The aim of this study was to measure quality of life (QOL) impairment in individuals currently suffering from Graves’ ophthalmopathy (GO) and to determine the correlation of GO-specific QOL scores with disease severity and activity.

**Subjects and methods:**

Seventy three GO-specific QOL surveys were prospectively analysed and compared with GO status. The GO-specific QOL survey was translated into Spanish and applied to Argentine patients with Graves’ disease (GD). Results were compared with presence or absence of GO, Clinical Activity Score (CAS), severity score, age, gender and thyroid function.

**Results:**

Fifty-six patients answered the survey and underwent complete ophthalmic evaluation, 15 did not have GO and were considered to be a control group. Appearance QOL score for patients with GO (53 ± 31.4) was lower than the control group (88.3 ± 17) (p < 0,000), no difference was observed in functional QOL score. There was a negative correlation between GO severity and both functional (*r* = -0.575; p < 0.000) and appearance QOL (*r* = -0.577; p < 0.000). Functional QOL differed between patients with active GO vs control group (p = 0.043). Patients with active and inactive GO had lower appearance QOL scores than control group (p < 0.000, p < 0.001 respectively).

**Conclusions:**

GO has significant impact on the life of these Argentine patients. QOL was worse in GO patients than in control group, functional QOL was mostly affected by the activity and appearance QOL was mainly altered by the effects of the disease. Patients with more severe GO had lower scores on both QOL scales.

## INTRODUCTION

Graves’ ophthalmopathy (GO) is a chronic, debilitating infiltrative eye disease that is clinically present in about 50% of patients with Graves’ disease. It is characterized by disfiguring proptosis, pain, redness and swelling of the eyelids, grittiness of the eyes and diplopia. Approximately 3% to 5% of patients suffer severe forms of the disease that could lead to permanent blindness (1,2). In addition, GO may be a severely disabling condition because of its effect on appearance and visual comfort.

Although the patient is usually the best person to monitor and judge outcomes in medical care, data concerning patients’ experiences about the disease and response to treatment are not routinely collected (3). Scoring standardized responses to standardized questions is an efficient way to measure health status and related QOL (4).

Therefore, Terwee and cols. (5) developed a GO QOL instrument for Dutch patients written in their native language which proved to be reliable among this population. The QOL questionnaire was used to evaluate the change in QOL scores associated with clinical improvement after different treatment modalities (6). Park and cols. (7) used the modified and translated GO-specific QOL survey in Australian patients and their results showed a significant correlation between impaired QOL score and disease severity. Using the Australian GO-GOL version, Wickwar and cols. (8) reported higher levels of potential cases of clinical anxiety and depression in GO than in other chronic diseases or facial disfigurements. GO-QOL visual function scores were explained by age, asymmetrical GO and depressed mood; and appearance scores were explained by gender, appearance-related cognitions and depressed mood. Choi and cols. (9) used the questionnaire among a Korean population and likewise found that the survey was associated with GO activity. Son and cols. (10), using TED-QOL, showed that age, soft-tissue inflammation and motility disorder had a positive correlation with overall and function-related QOL; while gender, soft-tissue inflammation and proptosis had a correlation with appearance-related QOL. Finamor and cols. (11) found seriously impaired scores on a 10-item health-related QOL questionnaire in Brazilian patients in the chronic and inactive stages of GO.

The aim of the study was to measure QOL impairment in individuals with GO (during the illness) and to determine the correlation of GO-specific QOL scores with disease severity and activity.

## SUBJECTS AND METHODS

### Design

We developed a cross-sectional study at our Endocrinology Division between June 2012 and December 2013. We translated the English version QOL survey provided by the EUGOGO (http://www.eugogo.eu/_downloads/clinical_evaluation/GO_QOL_EN.pdf) to Spanish. This questionnaire was distributed among Graves’ disease patients. A complete medical history and laboratory tests were performed. Patients were then referred for ophthalmological evaluation. The questionnaire was distributed after the medical evaluation, and returned completed by the patients before leaving the hospital.

### Subjects

The questionnaire was distributed to 95 Graves’ disease patients: 1) all recently diagnosed Graves’ disease patients (n = 36); 2) all patients prior to radioiodine treatment (n = 49) and 3) all patients with indication of systemic glucocorticoid treatment (n = 10 with severe and active GO and dysthyroid optic neuropathy-DON-).

GD diagnosis was performed on the basis of clinical features of thyrotoxicosis, elevated thyroid hormones, suppressed thyrotropin (TSH), positive serum TSH receptor antibodies (TRAb) and diffuse goiter. All patients provided informed written consent, and the institutional ethics committee approval was obtained before starting the study. Glucocorticoid treatment was prescribed for six patients with severe active GO (methylprednisolone 500 mg once weekly for 6 weeks, 250 mg once weekly for 6 weeks, total treatment period: 12 weeks) and four patients with sight-threatening GO (methylprednisolone 1g daily for 3 days).

Ophthalmologic examination was performed, in all cases, by the same professional. The evaluation included history of present illness, visual acuity, colour vision, pupillary examination, biomicroscopy, funduscopy and full orbital palpebral examination. An automated perimetry and an orbital CT scan were were performed in each case. GO was classified according to the Clinical Activity Score (CAS) and the severity score proposed by the EUGOGO modified consensus (Table 1) (12).

**Table 1:** EUGOGO modified Severity Score

Severity Score
Sight-threatening GO: patients with dysthyroid optic neuropathy (DON) or corneal ulceration. This category requires immediate intervention
Severe GO: patients without risk for vision, but the eye involvement has such an impact on their quality of life that justifies the risk of immunosuppressive therapy (if active) or surgery (if inactive)
GO moderate to severe: patients have one or more of the following: palpebral > 2 mm retraction, moderate or severe soft tissue involvement, exophthalmos > 3 mm above normal for race and gender, inconstant, or constant diplopia
GO mild: patients whose features of GO have only a minor impact on daily life insufficient to justify immunosuppressive or surgical treatment. They usually have only one or more of the following: minor lid retraction (< 2 mm), mild soft tissue involvement, exophthalmos < 3 mm above normal for race and gender, transient or no diplopia, and corneal exposure responsive to lubricants

### Methods

Answers on each subscale (functional and appearance) were transformed to scores ranging from 0 (worst) to 100 (best). We registered total QOL scores (average of functional and appearance scale), and also functional QOL scores and appearance QOL scores separately.

Serum total T3, total T4, free T4 and TSH were measured by chemiluminescence immunoassay (Immulite 1000, Siemens, USA). Serum TSH receptor antibodies (TRAb) concentrations were determined by RIA (RSR, UK). A value of TRAb ≥ 15% was considered positive. Normal reference ranges were free T4, 0.79 to 1.4 ng/dL; total T4, 5.0 to 11.5 ug/dL; total T3, 85 to 175 ng/dL; and TSH, 0.5 to 4.0 mUI/mL.

Statistical analyses were performed using the SPSS software package (version 17.0; SPSS Inc., Chicago, IL, USA). As a measure of reliability, internal consistency, based on item correlations within a subscale, was assessed by calculating Cronbach’s alphas. Continuous variables are reported as mean ± standard deviation (SD) or median (inter-quartile range). Student *t* test or one way ANOVA used for comparison of continuous variables. For correlations Pearson coefficient was used. Chi-square test was applied for comparing categorical variables. An alpha error of 0.05 was considered.

## RESULTS

**Figure 1:**
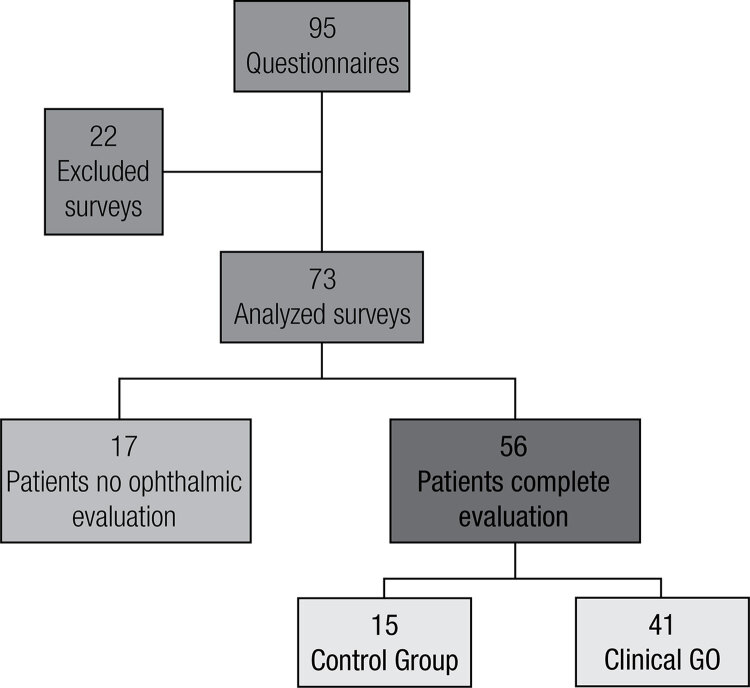
Population.

Of the 95 patients who received the questionnaire, 73 completed it adequately (77% response rate). Twenty-two surveys were excluded from analysis: 2 were not correctly identified, 1 survey contained more than 40% missing answers, 1 survey had more than 1 answer in one or more questions and 18 were not returned back to the treating physician (Figure 1). Of those who did not return the survey, 64.3% had no evidence of GO, significantly different to those who answered the questionnaire (26.8%; p = 0.01). Cronbach’s alphas were 0.79 for visual functioning and 0.90 for appearance.

Demographic characteristics of the 73 patients included are shown in Table 2. Fifty-six patients underwent complete ophthalmic evaluation, 15 of them did not have Graves’ orbitophathy and were considered to be a control group. The remaining 41 were considered to have clinical GO. The classification according to activity and severity of orbital disease is shown in Table 3. Mean CAS was 2.3 ± 1.8. There were no significant differences in disease severity and activity in relation to gender, thyroid functional status or age.

**Table 2:** Demographic characteristic of patients that completed de QOL questionnaire

Variable Value (n = 73)		
Age (years)		42.67 ± 15.2
Sex		
Female		80.8%
Male		19.2%
Smokers		40.7%
Thyroid function		
Hiperthyroidism		63.8%
Hiperthyroidism SC		21.8%
Euthyroidism		11.6%
Hipothyroidism		1.4%
Hipothyroidism SC		1.4%
Treatment		
Methimazole		
Titled		60.5%
Block/replacement		4.7%
No treatment		32.5%
Levothyroxine		4.9%
Previously treated with radioiodine		9.8%

**Table 3:** Characteristic of orbital disease in 41 patients with clinical GO

		Activity		Total
		Inactive	Active	
**Severity**	Mild	17	4	21
	Moderate	3	3	6
	Severe	4	6	10
	DON	0	4	4
	Total	24	17	41

The total mean QOL score over the 73 questionnaires was 67.8 ± 27.3, the means for functional and appearance scores were 67.5 ± 30.1 and 67.4 ± 31.9, respectively. There was a positive correlation between both functional and appearance scales (r = 0,567, p < 0,000). Functional and appearance scores were not significantly different between genders or with smoking or thyroid function (Table 4). There was not significant correlation between age and functional score (r = 0.19, p = 0.09.) or with appearance score (r = 0, p = 0.93).

**Table 4:** Functional and appearance scores according to demographic characteristics and ophthalmic evaluation

	Functional QOL	p	Appearance QOL	p
Sex		0.47		0.72
Female	65.6 ± 29.4		66.7 ± 32.6	
Male	72.1 ± 35.2		70 ± 29.8	
Smokers		0.58		0.32
Yes	66.7 ± 33.7		58.8 ± 33.9	
No	62.1 ± 30.3		67.7 ± 31.3	
Thyroid function		0.19		0.08
Hiperthyroidism	68.9 ± 28.8		72.8 ± 31.3	
Hiperthyroidism SC	73.6 ± 27.7		68.7 ± 31.3	
Euthyroidism	51.3 ± 38.1		46.2 ± 32.2	
Hipothyroidism*	18.7		18.7	
Hipothyroidism SC*	68.7		56.2	
Attend ophthalmic assessment		0.21		0.09
Yes	64.4 ± 32.1		62.5 ± 32.3	
No	74.9 ± 23.1		77.2 ± 26.9	
Orbitopathy				
Control group	75 ± 28.9		88.3 ± 17	
Severity	76.9 ± 23.7	**0.00**	66.3 ± 30.3	**0.00**
Mild				
Moderate	70.9 ± 16.5		40 ± 33.2	
Severe	40.9 ± 29.9		38.7 ± 29.4	
DON	6.8 ± 8.9		37.5 ± 8.8	
Activity				
Active	46.7 ± 34.3	**0.02**	47.3 ± 26.5	**0.00**
Inactive	69.2 ± 28.8		56.7 ± 34.3	

Functional and appearance scores are expressed as mean ± SD. * n = 1.

The frequency of responses to each question is shown in Table 5. The activities perceived as being most affected were: watching TV (50.8%), reading (46.5%) and doing something they wanted to do (40.9%). The majority of the participants considered that orbital disease altered their appearance (74.7%) or impaired their self-confidence (54.9%). Seventy percent of the patients felt that their visual function was affected in some way, and 79.5% considered the changes in their appearance to interfere with psychosocial function. When evaluating only the group of patients with GO (n = 41), 75.6% had altered visual function and 95.1% perceived a disturbance in their appearance. The appearance QOL score of the patients with GO (53 ± 31.4) was significantly lower than the control group (88.3 ± 17) (p < 0.000), but no difference was observed in functional QOL score (60.5 ± 32.6; 75 ± 28.9, respectively) (p = 0.161).

**Table 5:** Frequency of responses to each question of QOL survey

Visual functioning (%)	Severely limited	Little limited	Not limited	Missing response	Affected
Limitation in carrying out the following activity					
Q1. Riding a bike	12,7	14,1	45,1	28,2	26,8
Q2. Driving	8,5	14,1	42,3	35,2	22,6
Q3. Moving around the house	16,9	15,5	64,8	2,8	32,4
Q4. Walking outdoors	14,1	19,7	62	4,2	33,8
Q5. Reading	19,7	26,8	47,9	5,6	46,5
Q6. Watching TV	21,2	29,6	46,5	2,8	50,8
Q7. Hobbies or pastimes	16,9	15,5	52,1	15,5	32,4
Q8. Hindered from doing something they wanted to do	15,5	25,4	56,3	2,8	40,9

**Appearance (%)**	**Very much**	**A little**	**No**	**Missing response**	**Affected**

Q9. Changed appearance	43,7	31	22,5	2,8	74,7
Q10. Stared in the streets	22,5	15,5	62	-	38
Q11. People react unpleasantly	8,5	16,9	73,2	1,4	25,4
Q12. Influence on self confidence	22,5	32,4	42,3	2,8	54,9
Q13. Socially isolated	15,5	11,3	70,4	2,8	26,8
Q14. Influence on making friends	14,1	14,1	71,8	-	28,2
Q15. Appear less often on photos than before	28,2	11,3	60,6	-	39,5
Q16. Mask changes in your appearance	25,4	25,4	49,3	-	50,8

Frequency of responses to each question about visual function and psychosocial consequences as a result of changed appearance expressed as percentage in 73 patients.

The results of the questionnaire were not different between the patients who did not attend ophthalmic assessment (Functional QOL 74.9 ± 23.1; Appearance QOL 77.2 ± 26.9) and those who attended the ophthalmic appointment (Functional QOL 64.4 ± 32.1; Appearance QOL 62.5 ± 32.3) (p = 0.21 and p = 0.09, respectively).

### Results of the survey and GO activity

**Figure 2:**
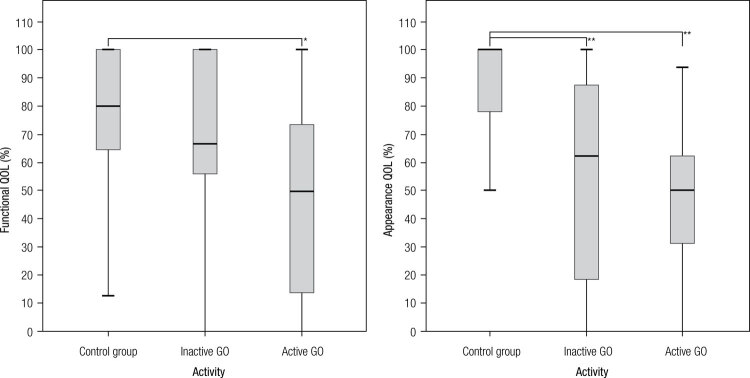
Differences between control group, inactive and active GO in functional and appearance QOL. * p < 0.05; ** p < 0.001.

There was significant difference in both functional (p = 0.026) and appearance (p < 0.000) QOL scores, among controls, inactive GO and active GO groups. Post hoc analysis for functional QOL showed statistically significant differences between patients with active GO vs the control group (p = 0.043). For appearance QOL, patients with active as well as inactive GO had lower scores than the control group (p < 0.000 and p < 0.001, respectively). No differences were observed between patients with inactive GO vs. those with active GO in this subscale (Figure 2).

In summary, functional QOL was mostly affected by the activity of the disease and appearance QOL was mainly altered just by having/bearing the orbital disease.

**Figure 3:**
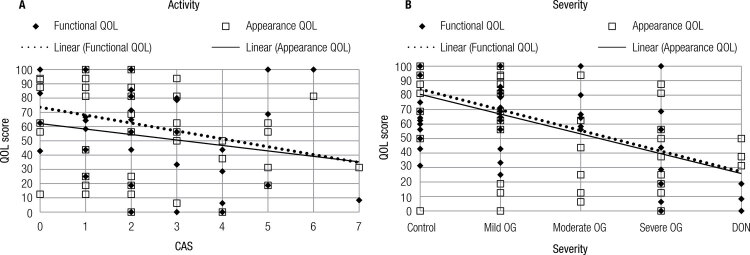
(A) Pearson Correlation between CAS and Functional QOL (r = -0.301; p = 0.056) and appearance QOL (r = -0.216; p = 0.176). (B) Pearson Correlation between Severity grades and Functional QOL (r = -0.575; p < 0.000) and appearance QOL (r = -0.577; p < 0.000).

There was no significant correlation between CAS and the QOL subscales, although there was a tendency for a negative correlation between CAS and functional QOL (p = 0.056) (Figure 3A).

### Results of the survey and GO severity

There was significant difference in both, functional (p < 0.000) and appearance (p < 0.000) QOL scores, between controls and the different degrees of GO.

**Figure 4:**
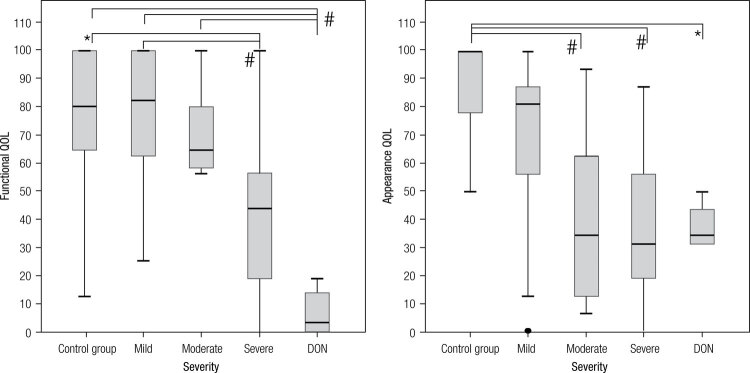
Differences between control group and severity grades in functional and appearance QOL. * p < 0.05; # p < 0.00.

Post hoc analysis showed that patients with DON had statistically lower functional QOL than patients with mild GO (p < 0.000), moderate GO (p = 0.008) and the control group (p = 0.001). Likewise, patients with severe GO had statistically lower functional scores than those with mild GO (p = 0.016) and controls (p = 0.039). In the appearance subscale, the difference was set between the control group and each one of other groups of patients, those with moderate GO (p = 0.014), severe GO (p = 0.001) and DON (p = 0.031) (Figure 4).

There was a negative correlation between the severity of GO and both functional and appearance QOL (p < 0.000) (Figure 3B).

Patients with indication of immunosuppressive therapy (those with severe and active GO and those with DON), had statistically lower values in both functional (p < 0.000) and appearance (p = 0.009) QOL scores compared with patients that did not require steroid treatment.

## DISCUSSION

The analysis of this survey in our population showed that more than two thirds of patients with GD (with and without GO) had functional and appearance affectation, regardless of age, sex and thyroid function. The alteration in appearance was present in virtually all patients with GO, with the appearance score significantly lower than controls. Although functional impairment was mentioned by 75.6% of patients with GO, the score was not different from patients without GO. This might be related to the considerable number of mild cases in this study. The functional scale was developed to evaluate the consequences of double vision and decreased visual acuity (5), which are only affected in more severe cases. Therefore, in our patients with DON and severe GO, the functional score was significantly lower than this score in patients with mild/moderate GO or without GO.

The present study is, to the best of our knowledge, the first that applied the GO-QOL questionnaire to a control group with GD without clinical GO. In this group of patients, 60% felt some impact on daily function and 46.6% felt psychosocial impairment. These results may be explained by the discomfort generated by other symptoms in patients with GD (13) or as a consequence of impaired general health perceptions and effects on quality of life.

The results of this survey in this group of patients from Argentina showed that GO has a significant impact on their lives, the majority of respondents reported limitation in daily activities such as reading and watching television, as well as impaired self-confidence and changes in appearance; similar to those reported by other authors in other countries (7-9). However, we had 35.2% missing answers for driving and 28.2% for cycling. These activities were reported as very frequently affected in other studies (7,14). We consider that this difference may be a consequence of the economic and social characteristics of our hospital populations with limited financial resources. Our patients often live far from their workplace and health care centres and in most cases they work for wages. We believe it would be useful to change these questions for others regarding the use of public transport and the loss of earning capacity, as it is known that GO is an important cause of occupational impairment (15).

Active GO patients were more physically impaired than those with inactive or no GO. Unexpectedly, we did not find statistical differences in the psychosocial area between patients with active vs inactive GO. The difference with other studies in a German (14) and a Korean (9) population could possibly be explained by the marked socio-cultural and economic differences of the populations as well as the small number of patients enrolled in this study.

We found no differences in functional and appearance scores regarding gender and age. Our results differ from those reported by Wickwar and cols. (8) since they found lower scores in functional QOL in elderly patients and lower scores in appearance QOL in women. The patients were recruited among those assessed to undergo orbital decompressive surgery, thus they included mostly cases with moderate to severe GO. In our sample, there was a high proportion of patients with mild disease, and this fact may partly explain the different results between both studies.

In this study, functional QOL was mostly affected by the activity of the disease and appearance QOL was mainly altered simply by the effects of orbital disease.

This study was started a year before the Spanish version of the questionnaire was available on the EUGOGO page, so we translated the questionnaire into Spanish. We assumed that the validity and internal consistency of our translation were well preserved as the Cronbach alphas of the Spanish language survey used in this study were comparable with those of the original Dutch GO-QOL survey (5) and those reported by other authors in English and Korean (7-9). The version currently available on the EUGOGO page is not the one we use, although it has no substantial differences with it.

Our study has some limitations: first, this was a cross-sectional study with a relatively small number of patients, which makes it hard to clarify a causal relationship; second, among patients who did not return the questionnaire, there was a significant number of patients without GO, which would, at some point, change the results of the control group; third, our results were derived from a relatively specific group of patients seen at a single academic institution, and the condition of patients in our clinic could be different from that of patients in the community setting.

In conclusion, GO has significant impact on the quality of life of these Argentine patients. QOL was worse in GO patients than in the control group, functional QOL was mostly affected by activity and appearance QOL was mainly altered simply by the effects of the disease. Patients with more severe GO had lower scores on both QOL scales.

To our knowledge, this is the first time this quality of life questionnaire for GO patients has been given to a Latin-American population. It is also the first study that compares Graves’ disease patients with and without clinical GO. This information will be useful for designing formal psychological support and treatment to help those with GO overcome their difficulties.
